# Smartphone-Based Meditation for Myeloproliferative Neoplasm Patients: Feasibility Study to Inform Future Trials

**DOI:** 10.2196/12662

**Published:** 2019-04-29

**Authors:** Jennifer Huberty, Ryan Eckert, Linda Larkey, Jonathan Kurka, Sue A Rodríguez De Jesús, Wonsuk Yoo, Ruben Mesa

**Affiliations:** 1 College of Health Solutions Arizona State University Phoenix, AZ United States; 2 Mays Cancer Center University of Texas Health MD Anderson San Antonio, TX United States; 3 College of Nursing and Health Innovation Arizona State University Phoenix, AZ United States

**Keywords:** mindfulness, meditation, smartphone, mHealth, cancer, quality of life

## Abstract

**Background:**

Myeloproliferative neoplasm (MPN) patients often report high symptom burden that persists despite the best available pharmacologic therapy. Meditation has gained popularity in recent decades as a way to manage cancer patient symptoms.

**Objective:**

The aim of this study was to examine the feasibility of 2 different consumer-based meditation smartphone apps in MPN patients and to examine the limited efficacy of smartphone-based meditation on symptoms compared with an educational control group.

**Methods:**

Patients (n=128) were recruited nationally through organizational partners and social media. Eligible and consented patients were enrolled into 1 of 4 groups, 2 of which received varying orders of 2 consumer-based apps *(10% Happier* and *Calm*) and 2 that received one of the apps alone for the second 4 weeks of the 8-week intervention after an educational control condition. Participants were asked to perform 10 min of meditation per day irrespective of the app and the order in which they received the apps. Feasibility outcomes were measured at weeks 5 and 9 with a Web-based survey. Feasibility outcomes were acceptability, demand, and limited efficacy for depression, anxiety, pain intensity, sleep disturbance, sexual function, quality of life, global health, and total symptom burden.

**Results:**

A total of 128 patients were enrolled across all 4 groups, with 73.4% (94/128) patients completing the intervention. Of the participants who completed the *10% Happier* app, 61% (46/76) enjoyed it, 66% (50/76) were satisfied with the content, and 77% (59/76) would recommend to others. Of those who completed the *Calm* app, 83% (56/68) enjoyed it, 84% (57/68) were satisfied with the content, and 97% (66/68) would recommend to others. Of those who completed the educational control, 91% (56/61) read it, 87% (53/61) enjoyed it, and 71% (43/61) learned something. Participants who completed the *10% Happier* app averaged 31 (SD 33) min/week; patients completing the *Calm* app averaged 71 (SD 74) min/week. *10% Happier* app participants saw small effects on anxiety (*P*<.001 *d*=−0.43), depression (*P*=.02; *d*=−0.38), sleep disturbance (*P*=.01; *d*=−0.40), total symptom burden (*P*=.13; *d*=−0.27), and fatigue (*P*=.06; *d*=−0.30), and moderate effects on physical health (*P*<.001; *d*=0.52). *Calm* app participants saw small effects on anxiety (*P*=.29; *d*=−0.22), depression (*P*=.09; *d*=−0.29), sleep disturbance (*P*=.002; *d*=−0.47), physical health (*P*=.005; *d*=0.44), total symptom burden (*P*=.13; *d*=−0.27), and fatigue (*P*=.13; *d*=−0.27). Educational control participants (n=61) did not have effects on any patient-reported outcome except for a moderate effect on physical health (*P*<.001; *d*=0.77).

**Conclusions:**

Delivering meditation via the *Calm* app is feasible and scored higher in terms of feasibility when compared with the *10% Happier* app. The *Calm* app will be used to implement a randomized controlled trial, testing the effects of meditation on symptom burden in MPNs.

**Trial Registration:**

ClinicalTrials.gov NCT03726944; https://clinicaltrials.gov/ct2/show/NCT03726944 (Archived by WebCite at http://www.webcitation.org/77MVdFJwM)

## Introduction

Myeloproliferative neoplasms (MPNs) are a rare blood cancer with the most common subtypes including polycythemia vera, essential thrombocythemia, and myelofibrosis. Those afflicted with MPN report a high prevalence of fatigue, anxiety, depression, pain, and sleep disturbance [1,2]. The vast majority of MPN patients (81% to 95%) report fatigue as the most prevalent and severe symptom reducing physical, social, and cognitive functioning as well as quality of life [2-4]. MPN patients often have a favorable life expectancy, with as many as 60% of patients living up to 15 years after diagnosis. Many patients even have the same life expectancy as the general population [5,6]. This means that MPN patients live much of their lives with fatigue, other symptoms, and the associated detrimental effects on overall functioning and quality of life [3,7].

There has been significant advancement in the pharmacological treatment of MPNs; however, for most patients, residual symptoms (ie, fatigue, anxiety, depression, pain, and sleep disturbance) persist, even with active pharmacologic therapy [8,9]. Additionally, standard-of-care treatments for MPN, including medication/drug therapy (ie, hydroxyurea), phlebotomy, and bone marrow transplant (the only curative therapy, reserved for those with severely reduced life expectancy), are associated with worsened symptoms and a reduced quality of life [10]. There is a need for research examining adjuvant, nonpharmacologic approaches (eg, mindfulness-based therapies) for MPN patients that may improve symptoms and that are not accompanied by negative side effects.

Extensive research in recent decades demonstrates the benefits of mindfulness-based therapies for alleviating fatigue, anxiety, depression, and sleep disturbance in both cancer and noncancer populations [11-16]. Mindfulness meditation has gained increasing attention as a complementary therapy for cancer patients, particularly for alleviating psychological comorbidities associated with cancer and its treatment [17]. Mindfulness meditation is the practice of moment-to-moment awareness in which the person purposefully focuses on the present moment without judgment [18]. Mindfulness meditation (hereinafter referred to as meditation) may be a potentially beneficial complementary therapy that improves MPN symptoms through increased nonjudgmental awareness of thoughts, feelings, and body sensations. These increases in awareness may help reduce feelings of anxiety and depression and may also help alleviate sleep disturbances resulting from sleep-interfering cognitive processes (ie, ruminating thoughts while trying to sleep) [12,16]. However, there has been minimal research investigating the effects of mindfulness meditation as a complementary therapy in hematological cancer patients and, specifically, no research conducted on MPN patients [19].

There has been a growing trend in recent decades toward digital (ie, Web-based and smartphone-based) interventions for health care access and to improve well-being. MPN patients may be a population that can greatly benefit from a digital intervention owing to (1) the rarity of MPNs, (2) the lack of specialized treatment centers in the United States, (3) the increasing prevalence of smartphone ownership in the United States, and (4) reported limitations to participation in in-person interventions among cancer patients. In a recent study, it was demonstrated that only 64% (18/28) and 29% (8/28) of MPN patients received their specialty care in the same state or city, respectively, in which they resided [20]. Additionally, the vast majority of Americans (more than 77%) own a smartphone, with 62% using their smartphones to get health information and 30% accessing a Web-based/mobile app to get educational content [21-23]. Finally, cancer patients report barriers to participating in in-person interventions, including fatigue, pain, and transportation/scheduling difficulties [24]. This asserts MPN patients as a population with the potential to benefit from digital meditation because they are more readily accessible and implemented with fewer resources than in-person meditation interventions.

As of January 2018, there were almost 6 million consumer-based smartphone apps available across both the Google Play Store and Apple’s App Store, the 2 leading app stores in the world [25]. Of these, there are over 500 mindfulness/meditation-based smartphone apps available [26]. Despite the large number of consumer-based smartphone mindfulness/meditation apps available, very little research has investigated the feasibility and efficacy of these apps. Components and features across meditation apps may be more feasible (ie, acceptable, demanded, and practical) than others, potentially impacting user outcomes such as frequency of use and long-term compliance [27]. Although research is limited investigating the desired features of a consumer-based smartphone meditation app, there are reports about desired features in physical activity smartphone apps (eg, automatic tracking of activity, tracking of progress toward goals, and integrated features) [28] and across health behavior change apps more broadly (eg, accuracy of information, security of personal information, effort required to track progress, and immediate effects on mood) that help to inform interventions [29]. However, no research has specifically investigated the feasibility and desirability of one meditation app compared with another, especially to inform future interventions in specific populations.

Owing to the potential beneficial effects that meditation may have on residual MPN symptoms, the potential ease of delivery to a rare cancer such as MPNs, and the potential for dissemination of a consumer-based smartphone meditation app across other cancer populations, there is a need to examine meditation delivered via consumer-based smartphone apps in MPN patients. Therefore, the aim of this study was to examine the feasibility of 2 different consumer-based meditation smartphone apps in MPN patients and to examine the limited efficacy of smartphone-based meditation on symptoms compared with an educational control group. According to Bowen et al [30], limited efficacy refers to testing the intervention with limited statistical power and is appropriate for feasibility studies.

## Methods

This study was approved by the Institutional Review Board at Arizona State University, and all participants signed an informed consent before participating in the study. All personal information was collected electronically and was stored in a password-protected computer at Arizona State University. The study was retrospectively registered with ClinicalTrials.gov.

### Study Design

This was a 4-group randomized controlled trial (RCT) with a cross-over design. A convenience sample of eligible and consented MPN patients was recruited and enrolled into 1 of 4 groups with varying order of receiving 2 consumer-based meditation smartphone apps (ie, *10% Happier* app or *Calm* app) in 4-week increments over 8 weeks. Group 1 received the *10% Happier* app followed by the *Calm* app; Group 2 received the *Calm* app followed by the *10% Happier* app; Group 3 received educational control followed by the *10% Happier* app; and Group 4 received educational control followed by the *Calm* app. We chose the 4-group design to maximize the number of participants who use both apps. We did not have a washout period because the primary goal was to assess feasibility, not efficacy.

We used the *Calm* app for this study because *Calm* is one of the most popular consumer-based mobile apps (ie, Apple’s app of the year in 2017), the lead author developed a relationship with *Calm* to conduct research using the app, and *Calm* agreed to provide the memberships to the app and share the tracking data with the research team without cost. The *10% Happier* app was chosen because they were one of the competing meditation mobile apps of *Calm* and they agreed to provide the memberships to the app and share the tracking data with the research team without cost. Additionally, both smartphone apps are available across all major smartphone platforms (ie, Android and iOS).

### Recruitment and Enrollment

MPN patients (n=128) were recruited online through MPN organizational partners with a flier outlining the study and its requirements. The study was advertised as a smartphone app meditation study. MPN patients interested in the study were asked to complete a Web-based eligibility questionnaire administered via Qualtrics. Textbox 1 describes the eligibility criteria. Researchers checked the eligibility questionnaires as they were completed and emailed patients their eligibility status. Eligible patients were invited to participate in a 20-min phone appointment in which the study details and informed consent were described in detail. MPN patients who completed the intake appointment were then sent an informed consent electronically via Qualtrics that included a place for their electronic signature. Ineligible patients received an email stating their ineligibility status as well as links to both consumer-based apps used in the study, in case the ineligible participant was interested in trying meditation.

Upon receipt of the completed informed consent, participants were randomly assigned to one of 4 groups. A group assignment list was computationally generated through randomizer.org by a research assistant to randomly allocate participants to one of 4 equally sized groups before study commencement This pre-generated list was then used by the same research assistant to place eligible, consented MPN patients into their group assignment in the order in which they consented to participate. Randomized participants were provided with a *welcome email* that contained (1) a welcome letter introducing them to the study, (2) a calendar detailing important study dates, and (3) instructions specific to the first assigned condition (ie, *CB* app, *Calm* app, or educational control) to be introduced for the first 4 weeks. After participants completed the first of their 2 4-week conditions (ie*,* *10% Happier* app, *Calm* app, or control), they were provided with another email that included instructions specific to their final condition (ie, *10% Happier* app, *Calm* app, or educational control).

**Eligibility criteria.**

Inclusion criteria:

- Had a diagnosis of essential thrombocythemia, polycythemia vera, or myelofibrosis identified by the treating physician
- Owned a mobile smartphone and were willing to download and use a meditation app (ie, *10% Happier* or *Calm*)
- Could read and understand English
- Were aged 18 years or older
- Were willing to be randomized to one of 4 groups: (1) *10% Happier* 4 weeks/*Calm* 4 weeks, (2) *Calm* 4 weeks/*10% Happier* app 4 weeks, (3) educational material 4 weeks/*10% Happier* app 4 weeks, and (4) educational material 4 weeks/*Calm* 4 weeks

Exclusion criteria:

- Engaged in ≥10 min/day of meditation on ≥5 days/week for the past 6 months
- Engaged in ≥60 min/week of tai chi, qigong, or yoga each week
- Utilized either the *10% Happier* app or the *Calm* app
- Resided outside of the United States

### Procedures

All participants were instructed to listen to the app’s introduction to meditation and, following this, were instructed to participate in a 10-min meditation each day, which they selected from the app’s library of meditations. The dose for the intervention was chosen because (1) the *Calm* and *10% Happier* app currently offer 10-min meditations (daily and series meditations), thus representing the length of time users are most likely to meditate using the app; and (2) to date, the ideal dose for mindfulness meditation interventions has not been established and effective mindfulness meditation interventions have ranged from 10-min sessions to 2-hour sessions and from one day per week to a daily practice [31-33]. Practical guidelines recommend that beginners start with short meditations lasting between 10 min and 30 min per session [34].

All study participants were asked to complete Web-based surveys at baseline, Week 5, and Week 9 via Qualtrics to assess feasibility outcomes (described in detail below), including study satisfaction and patient-reported outcomes. The survey consisted of 40 to 50 questions (depending on the condition the participant just completed and whether it was at baseline or post-condition) and was developed and tested internally among the research team for usability and functionality before use in the study. At the end of each 4-week condition, participants were emailed a unique survey link specific to their study identification number and were asked to complete the survey within 24 to 48 hours, if possible. Responses were collected electronically in Qualtrics and were then entered manually into a de-identified Excel spreadsheet for later data analysis. All participants received the same surveys in the same question order each time. The only difference between surveys was the wording of the satisfaction questions based on which condition they completed. Participants were able to scroll back through their survey responses by navigating back and forth through pages with a *back* button, giving them the flexibility to revise questions if they needed. Participants were only allowed to complete the survey once, and after it was submitted, they were not allowed access back into the survey.

Additionally, all study participants were instructed to wear a Fitbit (Fitbit Inc) device on their nondominant wrist throughout the 8-week intervention to measure physical activity and sleep; however, these data are not reported here. The intervention was 8 weeks because this is an appropriate length of time for a feasibility study in which the primary purpose was *not* to determine efficacy. Similar lengths have been used in other feasibility studies [30,35].

### Description of the Apps and the Control

#### 10% Happier App

The *10% Happier* app’s introduction to meditation incorporated basic information for those new to meditation. Daily meditations were selected from a library of meditations included within the app. Each of the meditations had a different focus (eg, grief, gratitude, choice, and letting go) and were approximately 10 to 12 min in length.

#### Calm App

The *Calm* app’s introduction to meditation incorporated basic educational information for those new to meditation while introducing brief experiential practices. Daily meditations were called the *Daily Calm* and were new and unique, provided by the app each day. The daily meditations had a different focus (eg, practicing patience, loving kindness, and gratitude) and were approximately 10 to 12 min in length. Meditations were also selected from a library of meditations with the app.

#### Educational Control

The control condition was provided with a 7-page educational handout that was developed by the research team before the study. The handout addressed MPN patient fatigue (eg, what causes fatigue?) as well as examples of and information related to evidence-based fatigue management strategies.

### Feasibility Outcomes

Feasibility (ie, acceptability, demand, and limited-efficacy testing) was defined according to Bowen et al [30]. Acceptability was measured with an investigator-developed set of satisfaction survey questions at Week 5 and Week 9 after participants completed each of their 4-week group assignments. Satisfaction questions were the same for all 3 conditions, with the exception of the wording that was altered slightly so that the question fit each condition. For example, a question asked in the satisfaction survey included: “How likely are you to continue using the (*insert condition here*) on a 1 to 5 scale (1 being very poor; 5 being excellent)”. Benchmarks for acceptability included ≥70% satisfaction with the apps’ content, ≥70% intending to continue using the app, ≥70% enjoying using the app, and ≥70% recommending it for other MPN patients. Demand was measured using adherence to the meditation intervention. Meditation participation was tracked by the smartphone app developers and reported weekly to the research team. Reports included (1) the date and time of each meditation participated in, (2) the title of the meditation, and (3) the duration of participation (ie, the time spent viewing the meditation) for each participant. Adherence benchmarks were defined as an average of ≥49 min/week of meditation across all participants (ie, ≥70% of prescribed meditation). There is little research suggesting appropriate benchmarks for acceptability and demand [30]. We based our benchmarks on a recently published methods paper for a National Institute of Health–funded study for the feasibility of a Web-based yoga intervention [36]. Patient-reported outcomes were assessed in all study participants via a Qualtrics questionnaire at baseline, Week 5, and Week 9. Demographics and MPN-related health history were included within the baseline questionnaire only. The National Institutes of Health Patient Reported Outcomes Measurement Information System (NIH PROMIS) and the MPN Symptom Assessment Form (MPN-SAF) were used to measure patient-reported outcomes. MPN-SAF measures included total symptom score and fatigue (Question 1 on MPN-SAF). NIH PROMIS measures included anxiety, depression, pain intensity, sleep disturbance, sexual function, global health, and quality of life (Question 2 on the global health scale). Table 1 describes each of these outcome measures.

**Table 1:** Summary of self-report outcome measures.

Measure, outcome		Scoring
**MPN SAF^a^**		
	Total symptom score	10 items (0-10 scale); total score range of 0-100 with higher score indicating worse symptom burden
	Fatigue	Item #1 from MPN SAF; 0-10. Scale with higher score indicating more fatigue
**NIH PROMIS^b^**		
	Anxiety	8-item measure with each question asked on a 1-5 scale; total cumulative raw score converted to standardized t-score; higher t-score represents more of the construct being measured
	Depression	8-item measure with each question asked on a 1-5 scale; total cumulative raw score converted to standardized t-score; higher t-score represents more of the construct being measured
	Pain intensity	3-item measure with each question asked on a 1-5 scale; total cumulative raw score converted to standardized t-score; higher t-score represents more of the construct being measured
	Sleep disturbance	8-item measure with each question asked on a 1-5 scale; total cumulative raw score converted to standardized t-score; higher t-score represents more of the construct being measured
	Sexual function	11-item measure with each question asked on a 1-5 scale; total cumulative raw score converted to standardized t-score; higher t-score represents more of the construct being measured
	Global health	10-item measure with each question asked on a 1-5 scale; total cumulative raw score converted to standardized t-score; higher t-score represents more of the construct being measured
	Quality of life	Item #2 on the Global Health scale; scored on a 1-5 scale with a higher raw score indicating a lower quality of life

^a^MPN-SAF: MPN Symptom Assessment Form.

^b^NIH PROMIS: National Institutes of Health Patient Reported Outcomes Measurement Information System.

### Data Analysis

Descriptive analyses were performed for baseline demographic characteristics using means and SDs of continuous data and using frequencies and proportions of discrete data for the 2 apps and 2 control groups, and dropouts. To test the limited efficacy of the intervention, a series of analysis of covariance analyses were performed for each of the NIH PROMIS outcomes using raw scores on measures of pain intensity, anxiety, depression, sleep disturbance, sexual function and discomfort indicators, global health, and quality of life. The analysis was adjusted for baseline PROMIS levels and covariates of the group membership (sequence effects), gender, education, and marital status. Race was not considered as a covariate as less than 5% of the sample was non-white Dunnett’s post hoc tested differences between treatment outcomes compared with baseline. A 2-tailed alpha error of .05 was the threshold for statistical significance. In addition, effect sizes (Cohen *d*) were calculated and classified as small (*d*=0.2), medium (*d*=0.5), and large (*d*=0.8) to examine differences at postcondition time. A negative effect size indicates the outcome measure decreased between the baseline and postcondition measure [37]. All analytical and visual evidence including appropriate statistics, *P* values, and graphs was reported using Statistical Analysis Software (SAS Institute, version 9.4) and Microsoft Excel (2016). A *P* value of <.05 was considered statistically significant.

## Results

### Recruitment and Enrollment

A total of 289 MPN patients were recruited and completed the eligibility survey between July 31 and October 18, 2017 (ie, two-and-a-half months). Of the 289 MPN patients who completed the eligibility survey, 33.2% (96/289) were ineligible and 44.2% (128/289) signed the informed consent and were enrolled into the study (see Multimedia Appendix 1). Eligible participants were enrolled into the study in the order they completed the eligibility questionnaire. A total of 73.4% (94/128) patients across all 4 groups completed the intervention. See Table 2 for protocol adherence and completion rates across groups. The results below are reported for participants who completed the intervention (ie, completed both postcondition surveys at Week 4 and Week 8).

**Table 2:** Protocol adherence.

Group	N (signed informed consent)	Completed post-Week 4 survey^a^, n (%)	Completed post-Week 8 survey^a^, n (%)
*10% Happier* followed by *Calm*	33	28 (85)	26 (79)
*Calm* followed by *10% Happier*	32	26 (81)	26 (81)
Control followed by *10% Happier*	35	24 (69)	24 (69)
Control followed by *Calm*	28	18 (64)	18 (64)
Total	128	96 (75)	94 (73)

^a^These surveys include satisfaction questions related to the completed 4-week group assignment.

There were no significant differences between groups on any demographic variable. Across all participants (n=128), average age was 58 (SD 12) years (*F*_1,126_=2.45; *P*=.12) and average body mass index was 27 (SD 6) kg/m^2^ (*F*_1,123_=.28; *P*=.60). The majority were female (104/128; χ^2^_3_=4.0; *P*=.26), white (123/128; χ^2^_9_=9.8; *P*=.36), well-educated with a bachelor’s education or higher (79/128; χ^2^_15_=15.7; *P*=.40), and married (95/128; χ^2^_15_=14.4; *P*=.49). In addition, the most common diagnosis among all participants was Essential Thrombocythemia (54/128) followed by Polycythemia Vera (48/128) and Myelofibrosis (26/128). Most participants had been diagnosed with their MPN for more than 3 years (81/128).

### Feasibility Outcomes

#### Acceptability

See Multimedia Appendix 2 for satisfaction survey responses. Of the participants who used the *10% Happier* app, 61% (46/76) enjoyed it, 66% (50/76) were satisfied with the content, and 77% (59/76) would recommend it to others. Of those who completed the *Calm* app, 83% (56/68) enjoyed it, 84% (57/68) were satisfied with the content, and 97% (66/68) would recommend it to others. Of those who completed the educational control, 91% (56/61) read it, 87% (53/61) enjoyed it, and 71% (43/61) learned something.

#### Demand

**Figure 1:**
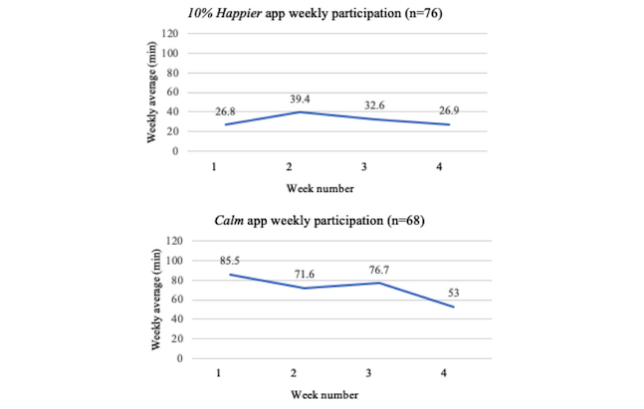
Weekly meditation participation.

Participants who completed the *10% Happier* app averaged 31 (SD 33) min/week and 30% (23/76) averaged ≥49 min/week of meditation, whereas participants who completed the *Calm* app averaged 71 (SD 74) min/week and 56% (38/68) averaged ≥49 min/week of meditation. See Figure 1 depicting average weekly meditation minutes completed by participants.

#### Limited Efficacy Testing

Mean differences in patient-reported outcomes from baseline to postcondition are reported in Multimedia Appendix 3. After completing 4 weeks of meditation using the *10% Happier* app (n=76), significant differences were found between baseline and postcondition for anxiety (*P*<.001; *d*=−0.43), depression (*P*=.02; *d*=−0.38), sleep disturbance (*P*=.01; *d*=−0.40), and physical health (*P*<.001; *d*=0.52). Mental health (*P*=.07; *d*=−0.30) and fatigue (*P*=.06; *d*=−0.30) were approaching significance. Although not statistically significant, a small-to-medium effect size and the 95% CI indicate differences in total symptom burden (*d*=−0.27; 95% CI −11.75 to −0.09).

After completing 4 weeks of meditation using the *Calm* app (n=68), significant differences were found between baseline and postcondition for sleep disturbance (*P*=.002; *d*=−0.47), vaginal discomfort (*P*=.03; *d*=−0.36), and physical health (*P*=.005; *d*=0.44). Small-to-medium effects and 95% CI indicated differences for measures of depression (*P*=.09; *d*=−0.29; 95% CI −3.49 to −0.14), total symptom burden (*P*=.13; *d*=−0.27; 95% CI −11.81 to −0.11), and fatigue (*P*=.13; *d*=−0.27; 95% CI −1.68 to −0.02), but were not significant.

After completing 4 weeks of the educational control (n=61), the only effect seen between baseline and postcondition was for physical health (*P*<.001; *d*=0.77).

## Discussion

### Principal Findings

The purpose of this study was to examine the feasibility of 2 different smartphone-based meditation apps in MPN patients and to examine the limited efficacy of smartphone-based meditation on symptoms compared with an educational control group. The findings of this study will inform the app to be used for a larger RCT to test for efficacy. We have identified the *Calm* app as being feasible for our future RCT as it met nearly all of our a priori feasibility criteria (ie, demand, acceptability, and limited-efficacy testing).

Our findings suggest that the *Calm* app had higher demand than the *10% Happier* app. Overall, the average weekly meditation across participants that completed *Calm* was greater than the prescribed 70 min/week and over half averaged ≥49 min/week of meditation (ie, demand cutoff criterion). Comparatively, those who participated in the *10% Happier* app only averaged 31 min/week of meditation and less than a third averaged ≥49 min/week of meditation. The adherence rates of the *Calm* app group are promising findings related to the potential for delivering meditation using a consumer-based smartphone app in MPN patients.

The adherence rates demonstrated by those completing *Calm* in this study are better than adherence rates of other smartphone-based meditation app studies [38,39]. A recent study conducted by Economides et al [38] investigated the effects of meditation delivered using the Headspace app on stress, affect, and irritability in novice meditators and found that participants (n=41) averaged approximately 44 min/week of meditation when asked to complete a total of 10 introductory 10-min meditations as they desired. Participants used the app for a short duration (approximately 16 days). Another recent study conducted by Bostock et al [39] that examined the effects of a 45-day Headspace meditation app intervention on work stress and well-being in healthy workers found that participants averaged approximately 42 min/week of meditation. Our study was 28 days in length (ie, the length participants were asked to use each app) and the *Calm* app group averaged approximately 71 min/week. It is not clear why our study had better participation in meditation as compared with the aforementioned studies. However, *Calm* may have characteristics (eg, app layout, presentation of content, and variety of content) within the app that make it more likely to be accepted and used by participants [28,29] when compared with Headspace or other meditation apps (ie, *10% Happier* app).

Those who completed *Calm* indicated that they enjoyed it (83%), were satisfied with the content (84%), and would recommend it to others (97%), each of which meets our benchmarks for acceptability. Of those that used the *10% Happier* app, 61% enjoyed it, 66% were satisfied with the content, and 77% would recommend to others. With the exception of those that would recommend it to others, the *10% Happier* app fell short of meeting benchmarks for acceptability. Self-report responses from those in *Calm* indicate the acceptability of the app compared with the *10% Happier* app. We conducted qualitative interviews related to the content and preferences for each of the apps; these data are being reported elsewhere [40]. However, briefly, the reasons *Calm* may have been more accepted than the *10% Happier* app were related to audio features (ie, the narrator voice was soothing and calming background sounds available) and other features such as pictures, stories, and a wide range of meditation topics.

Research with physical activity and other health behavior apps suggests automatic tracking of the specific activity, tracking of progress toward goals, and integrated features (eg, syncing with social media and connecting with music apps) are features most liked by users [28]. Both the *Calm* app and the *10% Happier* app share similarities in terms of their tracking features consistent with those reported in the literature as desired features of smartphone apps [28,29,41]. However, the *Calm* app automatically displays the tracking information following participation in meditation (*pops* up onto the screen) and includes minutes meditated, days in a row of mediating (ie, streak), and a calendar that highlights the days in which the app was used. To see the tracking of meditation in the *10% Happier* app, the user must navigate to their profile to look up the statistics that are displayed as days and minutes meditated. The differences in the way that the tracking is offered to the user may contribute to the differences in the acceptability of the apps. More exploration about the type of tracking and how participants prefer to interact with a tracking mechanism within a mobile app, specifically, a mediation app, is warranted.

Unlike the *10% Happier* app, *Calm* allows users to immediately share their meditation statistics (eg, number of meditations and time spent in meditation) through social media platforms (eg, Facebook) or text messages and emails (called *share status*). Even though we did not measure the use of the *share status*, the offering of this on the app may also contribute to its enhanced acceptability. Recent research has shown that participants like to share progress and that sharing progress updates and interacting within an online social platform may help increase feelings of accountability and may improve adherence rates in interventions [42,43]. In a recent qualitative study, participants with diverse health-related goals that shared updates on their progress via social media reported sharing their updates helped contribute to their accountability [42].

Both the *Calm* and *10% Happier* apps include a variety of content for users outside of just listening to meditation tracks. *Calm* includes sleep stories or sleep meditations to help users fall asleep. They also offer *Calm* Breathe, *Calm* Music, *Calm* Body, and relaxing scenes and nature sounds. *Calm* has masterclasses to educate participants about topics such as mindful eating, gratitude, and the importance of rest. The *10% Happier* app offers some similar content but is organized and delivered differently. For example, the content is organized on the bottom tool bar and users scroll to find the content they want to use (Calm organizes with a toolbar but also includes other screens for organization of content). The sleep content is only in the form of meditations (not sleep stories). Education for participants is in the form of courses with a meditation to follow-up the content (eg, a short 3 to 5 min lecture-based video followed by a meditation). On the basis of our findings, the way in which the *Calm* app organizes and delivers their content may be more appealing as compared with the *10% Happier* app. Future studies are warranted to determine what users specifically find most acceptable related to how content is organized and offered.

We observed limited efficacy on symptoms across both apps. Of note are the small effects observed in both apps on anxiety, depression, sleep disturbance, and total symptom burden. Although this is the first meditation study to be conducted in MPN patients, Huberty et al [20] identified that 12 weeks of Web-based yoga (60 min/week) had limited efficacy on anxiety, depression, sleep disturbance, and total symptom burden in MPN patients. Meditation and yoga differ in the sense that yoga includes a component of physical postures (asanas), but yoga does contain a meditation component and it is known that some of yoga’s benefits come from the meditation [44-48]. Meditation has been shown to improve anxiety, depression, and sleep disturbance symptoms in both cancer and noncancer populations through increased nonjudgmental awareness of thoughts, feelings, and body sensations [12,16]. Therefore, it is not surprising that this study showed limited efficacy in only 4 weeks on anxiety, depression, and sleep disturbances across both meditation apps. Studies of a longer duration may show larger effects. Our findings are preliminary in nature and RCTs powered for effectiveness are needed to determine the effects of consumer-based smartphone meditation on MPN patients.

### Limitations

There are limitations to this study that should be noted. First, our sample was disproportionately female (ie, 81% in this study vs approximately 53% female being typical of the MPN population [49]) and white (ie, 123/128, 96%). Second, we did not have a washout period in between each condition within the group assignments (ie, lack of washout time period between apps or between control and app conditions). As this was a feasibility study and we were not determining efficacy, a washout period was not necessary [50]. Finally, it is likely that the study findings represent those already motivated to use smartphones for health-related purposes. However, the literature suggests smartphone use is popular among cancer patients overall and that there is a large interest in accessing supportive care via smartphones in cancer patients [51,52]. Importantly, future studies could determine the effects of a mobile app to improve health-related outcomes in cancer patients, especially when cancer patients are already using mobile apps.

### Conclusions

Delivering smartphone meditation via the *Calm* app is feasible as it met most of our feasibility criteria (ie, demand, acceptability, and limited-efficacy testing) and scored higher in terms of feasibility when compared with another consumer-based app (ie, *10% Happier* app). Future RCTs are needed to examine meditation with the *Calm* app and its effects on MPN patient symptoms. The findings of this study will be used to inform the development of a National Institutes of Health R01 grant app for an RCT examining the efficacy of *Calm* on MPN patient symptoms.

## Appendix

Multimedia Appendix 1 Enrollment.

Multimedia Appendix 2 Satisfaction Survey Responses.

Multimedia Appendix 3 Patient-Reported Outcomes.

## Abbreviations

MPN: myeloproliferative neoplasm
MPN-SAF: Myeloproliferative Neoplasm Symptom Assessment Form
NIH PROMIS: National Institutes of Health Patient Reported Outcomes Measurement Information System
RCT: randomized controlled trial
