# Supplementary figures and images for: Smartphone-Based Meditation for Myeloproliferative Neoplasm Patients: Feasibility Study to Inform Future Trials
Source: JMIR Form Res. 2019 Apr 29;3(2):e12662. doi: 10.2196/12662 (PMC6658299; doi:10.2196/12662)

## Appendix 1. Enrollment

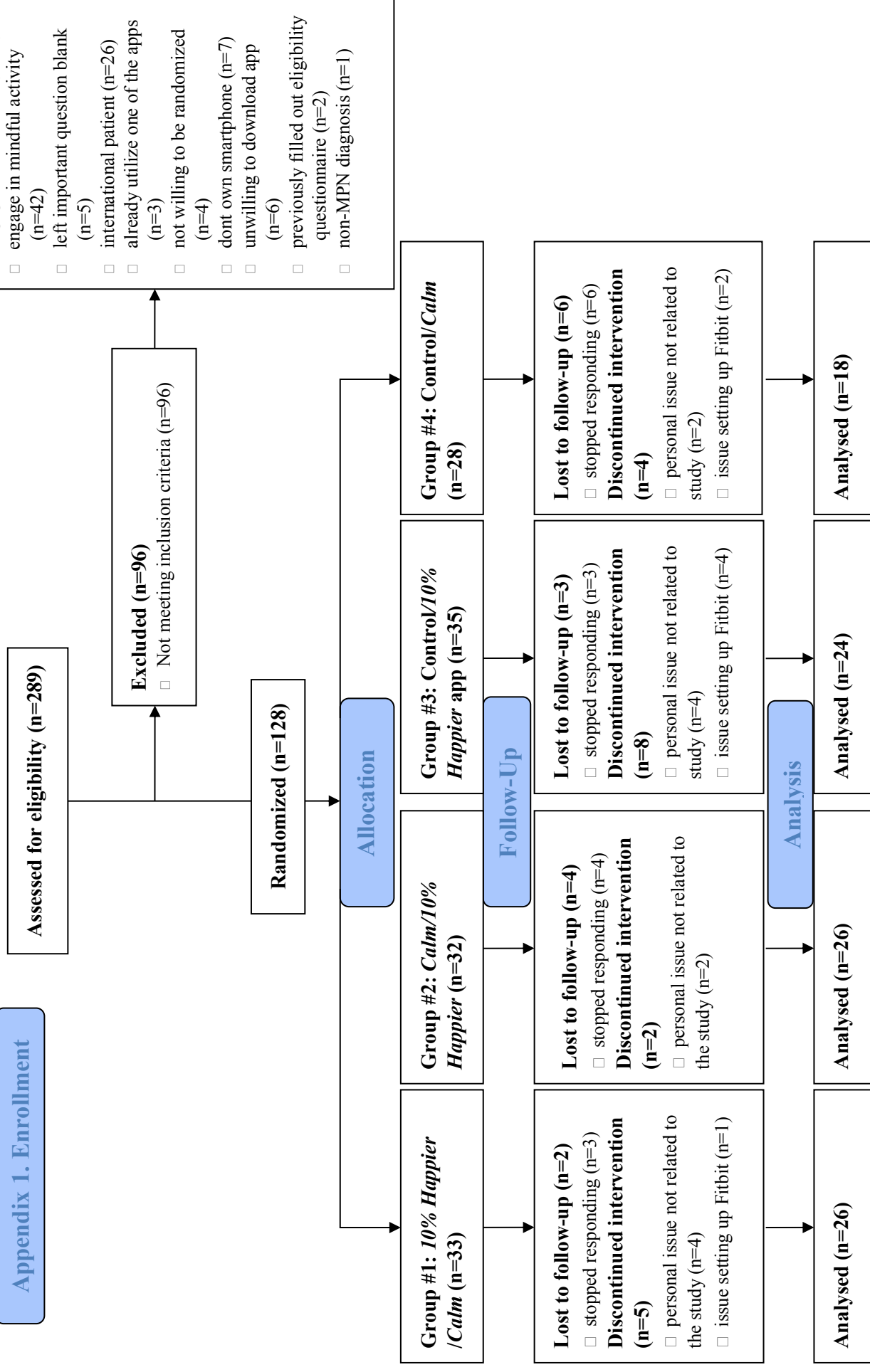

Supplement: Multimedia Appendix 1 [file formative_v3i2e12662_app1.pdf]
