# Supplementary material for: Smartphone-Based Meditation for Myeloproliferative Neoplasm Patients: Feasibility Study to Inform Future Trials
Source: JMIR Form Res. 2019 Apr 29;3(2):e12662. doi: 10.2196/12662 (PMC6658299; doi:10.2196/12662)
Supplement: Multimedia Appendix 2 [file formative_v3i2e12662_app2.pdf]

**Appendix 2. Satisfaction survey responses**	***All Calm Users***	***All 10% Happier Users***	***Two-App Users***		***One-App Users***	
	***Calm* (n=63)**	***10% Happier* (n=67)**	***Calm* (n=51)**	***10% Happier* (n=46)**	***Calm* (n=12)**	***10% Happier* (n=21)**
Question 1: On a scale of 1-5 (1=did not enjoy at all, 5=very much enjoyed), how would you rate your overall enjoyment with using the meditation app?						
(1) did not enjoy at all	1.6%	0.0%	2.0%	0.0%	0.0%	0.0%
(2)	3.2%	10.5%	3.9%	15.2%	0.0%	4.8%
(3) neutral	12.7%	28.4%	9.8%	26.1%	16.7%	38.1%
(4)	41.3%	23.9%	45.1%	23.9%	25.0%	14.3%
(5) very much enjoyed	41.3%	37.3%	39.2%	34.8%	58.3%	42.9%
Question 2: How would you rate your overall satisfaction with the content of the meditation app?						
(1) very satisfied	33.3%	35.8%	31.4%	32.6%	50.0%	47.6%
(2) satisfied	50.8%	29.9%	52.9%	30.4%	33.3%	19.0%
(3) neither satisfied nor dissatisfied	14.3%	31.3%	13.7%	32.6%	16.7%	33.3%
(4) dissatisfied	1.6%	3.0%	2.0%	4.3%	0.0%	0.0%
(5) very dissatisfied	0.0%	0.0%	0.0%	0.0%	0.0%	0.0%
Question 3: Would you recommend using the meditation app for MPN cancer patients?						
(1) yes	96.8%	76.9%	96.1%	73.9%	100.0%	85.7%
(2) no	3.2%	23.1%	3.9%	26.1%	0.0%	14.3%
Question 4: How likely are you to continue using the meditation app in the future?						
(1) extremely likely	28.1%	16.4%	23.5%	15.2%	50.0%	19.0%
(2) likely	48.4%	38.8%	56.9%	37.0%	16.7%	38.1%
(3) neutral	12.5%	16.4%	5.9%	15.2%	33.3%	23.8%
(4) unlikely	7.8%	20.9%	9.8%	21.7%	0.0%	19.0%
(5) extremely unlikely	3.1%	7.5%	3.9%	10.9%	0.0%	0.0%
