# Supplementary material for: Smartphone-Based Meditation for Myeloproliferative Neoplasm Patients: Feasibility Study to Inform Future Trials
Source: JMIR Form Res. 2019 Apr 29;3(2):e12662. doi: 10.2196/12662 (PMC6658299; doi:10.2196/12662)
Supplement: Multimedia Appendix 3 [file formative_v3i2e12662_app3.pdf]

Appendix 3. Mean differences in patient-reported outcomes between baseline and post-condition time points.

Outcome	n[a]	Condition	Mean[b]	SE	95% CI		Statistic (df)		Value	*P*-value[c]	Effect Size
Pain Intensity	319						*F*(3,306)	=	0.29	>0.05	
		Baseline	6.70	0.32	6.08	7.33					
		Difference at Post-Condition:									
		post-10% Happier	-0.28	0.41	-1.10	0.53	*t*(306)	=	-0.68	.86	-0.09
		post-*Calm*	-0.32	0.42	-1.14	0.49	*t*(306)	=	-0.78	.81	-0.11
		post-control	-0.02	0.48	-0.97	0.92	*t*(306)	=	-0.05	.99	-0.01
Anxiety	317						*F*(3,305)	=	3.51	.02	
		Baseline	17.14	0.73	15.70	18.58					
		Difference at Post-Condition:									
		post-10% Happier	-3.02	0.95	-4.88	-1.16	*t*(305)	=	-3.19	.00	-0.43
		post-*Calm*	-1.51	0.95	-3.38	0.35	*t*(305)	=	-1.60	.29	-0.22
		post-control	-1.14	1.10	-3.30	1.03	*t*(305)	=	-1.03	.64	-0.16
Depression	319						*F*(3,306)	=	3.29	.02	
		Baseline	14.28	0.65	13.00	15.55					
		Difference at Post-Condition:									
		post-10% Happier	-2.38	0.85	-4.05	-0.72	*t*(306)	=	-2.81	.02	-0.38
		post-*Calm*	-1.81	0.85	-3.48	-0.14	*t*(306)	=	-2.13	.09	-0.29
		post-control	-0.38	0.99	-2.32	1.56	*t*(306)	=	-0.39	.97	-0.06
Sleep Disturbance	319						*F*(3,306)	=	5.39	.001	
		Baseline	22.6286	0.779	21.096	24.1615					
		Difference at Post-Condition:									
		post-10% Happier	-3.08	1.02	-5.08	-1.08	*t*(306)	=	-3.03	.01	-0.40
		post-*Calm*	-3.53	1.02	-5.54	-1.53	*t*(306)	=	-3.46	.002	-0.47
		post-control	-1.01	1.18	-3.34	1.32	*t*(306)	=	-0.85	.76	-0.13
Erectile Function	62						*F*(3,52)	=	1.92	.14	
		Baseline	6.34	0.48	5.38	7.30					
		Difference at Post-Condition:									
		post-10% Happier	0.44	0.73	-1.03	1.90	*t*(52)	=	0.60	.86	0.08
		post-*Calm*	-0.91	0.73	-2.38	0.55	*t*(52)	=	-1.25	.43	-0.17
		post-control	0.14	0.73	-1.32	1.60	*t*(52)	=	0.19	.99	0.03
Sexual Activity Interest	319						*F*(3,306)	=	0.77	.51	

		Baseline	4.79	0.24	4.32	5.26				
		Difference at Post-Condition:								
		post-10% Happier	-0.05	0.31	-0.67	0.57	*t*(306) =	-0.15	.99	-0.02
		post-*Calm*	-0.41	0.32	-1.03	0.22	*t*(306) =	-1.28	.47	-0.17
		post-control	0.17	0.37	-0.55	0.89	*t*(306) =	0.47	.95	0.07
Vaginal Discomfort	123						*F*(3,245) =	2.37	.07	
		Baseline	4.32	0.41	3.52	5.12				
		Difference at Post-Condition:								
		post-10% Happier	-0.45	0.47	-1.37	0.47	*t*(245) =	-0.96	.69	-0.13
		post-*Calm*	-1.21	0.46	-2.11	-0.31	*t*(245) =	-2.65	.03	-0.36
		post-control	-0.55	0.53	-1.59	0.50	*t*(245) =	-1.03	.64	-0.16
Satisfaction w/ Sex Life	283						*F*(3,304) =	1.43	.23	
		Baseline	6.28	0.45	5.41	7.16				
		Difference at Post-Condition:								
		post-10% Happier	0.41	0.58	-0.73	1.56	*t*(304) =	0.71	.85	0.09
		post-*Calm*	-0.95	0.59	-2.11	0.21	*t*(304) =	-1.62	.28	-0.22
		post-control	-0.05	0.68	-1.38	1.28	*t*(304) =	-0.07	.99	-0.01
Orgasm	315						*F*(3,302) =	0.99	.40	
		Baseline	2.70	0.21	2.28	3.12				
		Difference at Post-Condition:								
		post-10% Happier	-0.25	0.28	-0.80	0.30	*t*(302) =	-0.88	.74	-0.12
		post-*Calm*	-0.45	0.28	-1.01	0.10	*t*(302) =	-1.60	.29	-0.22
		post-control	0.01	0.32	-0.62	0.65	*t*(302) =	0.05	.99	0.01
Physical Health	316						*F*(3,303) =	11.5	<.001	
		Baseline	10.88	0.29	10.31	11.45				
		Difference at Post-Condition:								
		post-10% Happier	1.47	0.38	0.73	2.21	*t*(303) =	3.91	<.001	0.52
		post-*Calm*	1.23	0.38	0.47	1.98	*t*(303) =	3.20	.005	0.44
		post-control	2.18	0.44	1.32	3.05	*t*(303) =	4.98	<.001	0.77
Mental Health	315						*F*(3,302) =	2.4	.07	
		Baseline	11.78	0.39	11.01	12.55				
		Difference at Post-Condition:								
		post-10% Happier	-1.15	0.51	-2.15	-0.16	*t*(302) =	-2.28	.07	-0.30

		post-*Calm*	-0.93	0.51	-1.92	0.07	*t*(302)	=	-1.83	.18	-0.25
		post-control	0.03	0.58	-1.12	1.18	*t*(302)	=	0.05	.99	0.01
Total Symptom Burden	307						*F*(3,294)	=	2.31	.08	
		Baseline	42.32	2.29	37.82	46.83					
		Difference at Post-Condition:									
		post-10% Happier	-5.92	2.96	-11.75	-0.09	*t*(294)	=	-2.00	.13	-0.27
		post-*Calm*	-5.96	2.97	-11.81	-0.11	*t*(294)	=	-2.01	.13	-0.27
		post-control	0.54	3.46	-6.26	7.34	*t*(294)	=	0.16	.99	0.02
Fatigue	317						*F*(3,304)	=	2.71	.05	
		Baseline	6.60	0.32	5.97	7.24					
		Difference at Post-Condition:									
		post-10% Happier	-0.95	0.42	-1.78	-0.13	*t*(304)	=	-2.27	.07	-0.30
		post-*Calm*	-0.85	0.42	-1.68	-0.02	*t*(304)	=	-2.00	.13	-0.27
		post-control	0.13	0.49	-0.84	1.10	*t*(304)	=	0.26	.99	0.04

Controlling for gender, education level, and marital status

[a] n of PROMIS outcome measures

[b] Baseline Condition shows mean, SE for raw scores, Post-treatment Conditions shows mean difference, SE of mean difference

[c] Dunnett-Hsu adjustment for comparison of multiple groups
